# Use and Diversity of Palm (Arecaceae) Resources in Central Western Brazil

**DOI:** 10.1155/2014/942043

**Published:** 2014-01-02

**Authors:** Renata Corrêa Martins, Tarciso de Sousa Filgueiras, Ulysses Paulino Albuquerque

**Affiliations:** ^1^University of Brasília, Brasília, CP 4457, 70919-990 Brasilia, DF, Brazil; ^2^Institute of Botany, São Paulo, CP 3005, 01061-950 São Paulo, SP, Brazil; ^3^Federal Rural University of Pernambuco, Laboratory of Applied and Theoretical Ethnobiology (LEA), Rua Dom Manoel de Medeiros, s/n, Dois Irmãos, 52171-900 Recife, PE, Brazil

## Abstract

Arecaceae Schultz-Sch. (Palmae Juss.), a member of the monocotyledon group, is considered one of the oldest extant angiosperm families on Earth. This family is highly valuable because of its species diversity, its occurrence in a wide range of habitats, and its status as an integral part of the culture and the family-based economy of many traditional and nontraditional communities. The main objectives of this study were to perform an ethnobotanical study involving these palms and a “Quilombola” (Maroon) community in the municipality of Cavalcante, GO, Brazil. The variables gender, age, and formal schooling had no influence on the number of species recognized and used by the Kalungas. Ethnobotanical studies based on traditional knowledge in addition to use and management of palms are fundamental aspects for planning and appliance of public policies directed to the use of natural resources and improvement of life quality.

## 1. Introduction

Several studies of palm species have highlighted their variety of uses. Palms have often been cited as useful materials for construction, food, handcrafts, rituals, and therapeutics. Quantitative methods have been used in ethnobotanical studies to analyze the knowledge and use of palms by local people, with the work of Bates [1], Byg and Balslev [2], Byg et al. [3], Campos and Ehringhaus [4], Nascimento et al. [5], and Zambrana et al. [6] deserving special mention.

The family Arecaceae offers a high potential value for the Brazilian Cerrado because it has great species diversity (approximately 60 species) [7], exists in all types of habitat (from forest to savanna and grassland) [8], and is part of the culture and family-based economies of many traditional and non-traditional communities of the Cerrado. However, few ethnobotanical studies have been conducted on the use of palms, despite the extensive palm groves and the cultural diversity of the region (see [5, 9, 10]).

The Brazilian Cerrado is also home to a diverse range of Quilombola (Maroon) communities, who represent the descendants of African people who arrived in Brazil as slaves during the colonial period. Ethnobotanical and ethnopharmacological research has been conducted in Quilombola communities with an emphasis on medicinal plants, including studies by Amoroso [11], Rodrigues and Carlini [12], Franco and Barros [13], Rodrigues [14], and Crepaldi and Peixoto [15]. However, because of the wide distribution of these communities across Brazil and their diverse cultures, landscapes, and natural resources, a great deal of ethnobiological knowledge remains unexplored.

This study aims to add to the local botanical knowledge regarding the family Arecaceae (Palmae) in Brazil, especially in the Cerrado, by studying communities closely linked to the Cerrado and its natural resources to understand their relationships with and the use of native palms. This paper aims to answer the following questions.

- What is the diversity of palms known and used by a Quilombola community of the Brazilian Cerrado?
- Do the gender, age, and education of the respondents influence their recognition and use of palm species?
- Which palms are the favorites of the local community, and which parts of those palms are the most important?

## 2. Materials and Methods

### 2.1. Area of the Study

The field work was conducted in a Quilombola community in Goiás State. The population of Goiás is approximately 1,847,671 (married people over 10 years old) [16], distributed among 246 cities. The 73 Quilombola communities in Goiás are distributed among 24 cities. Cavalcante is home to the greatest number of Quilombola communities (23), followed by Monte Alegre (15) and Teresina de Goiás (6) [17]. People from the Quilombola communities in these three cities are called Kalungas or Calungas.

For the Kalunga people, the name Kalunga refers to a stream that passes near the Contenda farmland. In the past, many people did not like to be referred to as Kalunga because it was synonymous with witchcraft and laziness. However, many became proud of this identity when their presence on the land became legalized [18, 19]. The Kalunga territory was established by Africans and African-Brazilians who escaped from slavery and remained in the region after the decline of mining and the abolition of slavery. The freed slave population from other areas also migrated to the region looking for land to cultivate to enable an autonomous and free existence [20].

The Kalungas lived for 200 years isolated in the valleys of the tributaries of the Parana river and on the edges of the “Chapada dos Veadeiros” and formed various groups, with four main large groups located in Vão de Almas, Contenda, Vão do Moleque, and Ribeirão dos Bois and other smaller groupings located in Riachão, Sucuri, Saco Grande, Volta do Canto, Olha d'água, Ema, Taboca, Córrego Fundo, Terra Vermelha, Lagoa, Porcos, Brejão, Fazendinha, Vargem Grande, Engenho II, Funil, Capela, and a dozen other places [19].

The Kalungas were scientifically discovered in 1982 by the anthropologist Mari de Nazaré Baiocchi. Prior to this time the Kalungas practiced subsistence living, planting to eat and exchanging products such as cassava flour, leather, and mangaba for fabrics, salt, and other supplies in neighboring towns. The “discovery” of the Kalungas led them to obtain the legal right to own their land, preventing land invaders from displacing them [21].

The area of the Historic Kalunga Site was registered by Law No. 11.406 on January 21, 1991, and regulated by Law No. 19 on January 5, 1996. A large part of this scenic territory consists of mountains, with extensive steep areas, large valleys, and many rivers. Only 30% of this area is arable [19], and accessing many communities is difficult.

The area of the Kalunga territory is a well-preserved Cerrado region, especially in areas with steep topographies featuring hillsides and mountain ranges. The area features extensive palm groves and valleys, and most of the communities are located near rivers, creeks, and streams. Their houses are constructed from adobe, with palm leaf roofs and packed earth floors. These communities practice simple lifestyles, for example, using wood stoves. Some communities (Engenho II and Vão do Moleque) have received benefits from the federal government, and many residents in those communities have acquired new homes built with masonry and with clay roofs.

The social organization of these communities is typically centered around family relationships [18]. The family is the basic unit of society, in which people help each other and the ethical and cultural values of the community are passed on. These communities have cycles of events based on the planting and harvesting times. The Cerrado features two defined seasons (wet summer and dry winter), and these seasons determine the time spent cultivating the land [19, 22].

The Kalunga community lives by subsistence agriculture, including husbandry of cattle, pigs, and poultry and extraction of Cerrado fruits (watermelon, banana), vegetables, and medicinal plants cultivated in home gardens and orchards [22–24]. The main crops are corn (90% of families) followed by beans (80%), cassava (70%), rice (50%), pumpkin (50%), and sugar cane (40%) [25].

Approximately 60% of residents use cassava to produce flour, which is one of the most economically important products for these communities [25]. Kalunga gardens are strategically important due to their growth and maintenance of a wide variety of plants [22].

**Figure 1:**
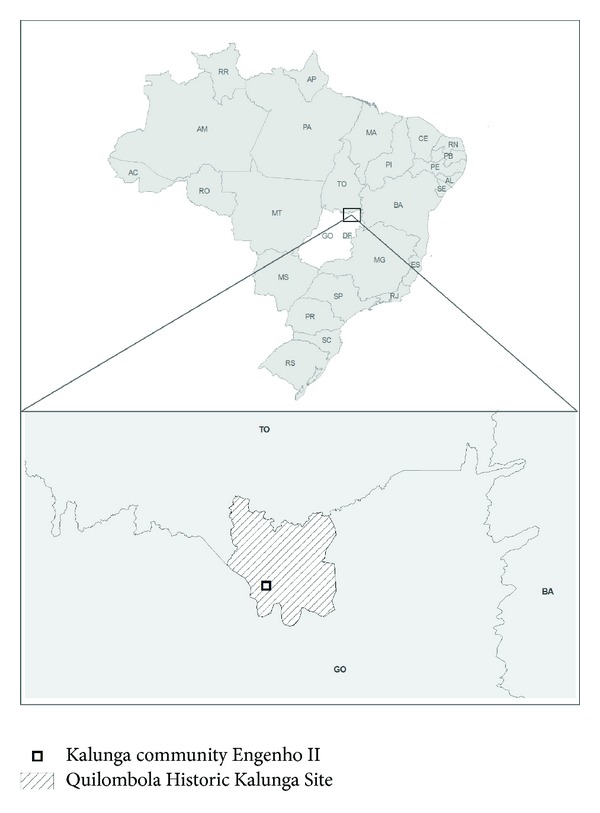
Map of Brazil denoting Goiás State (GO), the Quilombola Historic Kalunga Site, and the Kalunga Community Engenho II in Cavalcante, GO, central western Brazil.

The Kalunga territory borders Tocantins state, occupying an area of approximately 253.2 thousand hectares at the following geographic coordinates: from 13°20′ to 13°27′ south latitude and from 47°10′ to 47°20′ west latitude from Greenwich. This territory is located in the Chapada dos Veadeiros area, northeast of Goiás state, 600 km from Goiânia and 330 km from Brasilia. The region borders the following cities: Monte Alegre de Goiás, Terezina de Goiás, and Cavalcante (Figure 1).

This study examines the Kalunga community Engenho II. This community is located 27 km from the municipality of Cavalcante and is accessed via an unpaved road that reaches the center of the community (13° 34′ 57′′ S and 47° 28′ 21′′W). The houses of the residents are built of mixed materials, including adobe and masonry, and have roofs made of buriti leaves and clay tiles. At the end of this research, the community consisted of approximately 100 homes and approximately 550 residents.

The residents of the Engenho II practice subsistence agriculture and participate in the regional market, eventually as employees, selling or exchanging products. The cultivated areas are far from the farmhouses of the community. Some residents engage in a small amount of commerce by selling snacks and preparing meals to order. Some Kalungas are public employees and several are tour guides. One of the main income-generating activities in this community is the rural tourism, with attractions including waterfalls, hiking, and homemade food.

### 2.2. Ethnobotanical Data Collection

The process of requesting access to study this traditional community was filed in September 2009 with the request of a signature to provide prior consent. Resolution No. 250 was published in the Official Gazette on April 16, 2010. The process number that authorized this research is 02000.002793/2009-73 and the authorization number is 48/2009.

This work was performed in three phases.

#### 2.2.1. Phase I: Informal Interviews

To develop a preliminary list of palm species known among the residents of the Engenho II community, approximately 20 residents were interviewed, including some tourist guides and local leaders. The interviews took place in a shed used to provide tourist services. We used a diary to record the names of the palms and the locations of the populations, along with information about the use of the species and any other interesting details. In this step, 16 palm species were recognized.

#### 2.2.2. Phase II: Guided Tour and Systematic Botany Collection

A guided tour was conducted to support and validate the names of the plants mentioned in the informal interviews [26]. In this step, a floristic survey of all palm species in the region was conducted. Samples were collected from sites where palms occurred. Areas with forest, savanna, and grassland formations were examined, and all 16 species mentioned during the informal interviews were photographed, collected, and identified.

Jorge Moreira da Silva, Damião Santos Rosa, and João Francisco Maia participated in the botanical collection expeditions. The specimens were deposited in the UB (University of Brasilia) herbarium. Due to difficulties in collecting some of these palm species, some were identified in the field, while others were determined according to the literature [27–29].

#### 2.2.3. Phase III: Sample Selection

This study considered all local residences (*n* = 100), 12 of which had no residents. We visited the community 12 times, with each visit lasting 2–4 days, beginning in May 2010 and ending in February 2011. The preferred informant was the oldest resident of the house present at time of the visit.

The ethnobotanical data were collected in a semi-structured interview conducted using the checklist/interview method [26, 30]. This technique is considered the best option when there is only one chance to interview the informant [26]. In the first part of the interview, the questions determined the socioeconomic profile of the informant such as gender, age, and education. In the second part, the questions referred directly to the recognition and use of palms, as described below.

Eighty-eight individual interviews were conducted; 56 of the interviewees were women and 32 were men, with ages ranging between 18 and 82 years. The main activity performed by the residents is the cultivation of land (farming) (70; *n* = 88), and men and women perform other activities, including domestic duties and rural tourism. The respondents also included one health officer, one public employee, two school cooks, two teachers, and three local business owners.

Visual stimuli enable an evaluation of the respondent's knowledge and use of plants (see [30–32]). Considering the size of palm samples, photographs were the best visual stimuli for this study. The systematic use of photographs of plants in ethnobotanical interviews is still poorly represented in the literature [30, 33–35], but this approach has been shown to be more effective than the use of voucher specimens [36].

**Figure 2:**
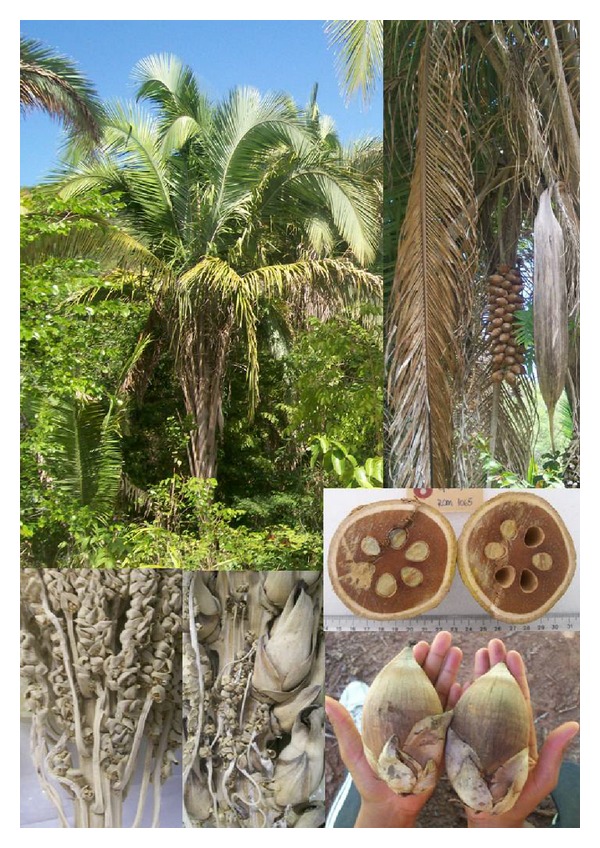
A figure used during the interviews in the Kalunga community Engenho II, Cavalcante, GO, central western Brazil. *Attalea speciosa* (coconut palm).

Sixteen A4-sized photographs of each palm species present in the region were prepared (Figure 2). The photographs show the plant in its habitat and the leaf, flower, and/or fruit shapes. A preliminary compilation of the figures was used in a pilot formulary in the community to determine whether the photographs accurately captured the true plant morphology and fit with local knowledge. Thus, it was possible to ensure that “the informants and the interviewer were talking about the same plant” [33].

In each residence, the figures were handed to the informant one at a time. Then, for each species shown, the following questions were asked: Do you know this plant? Have you ever used it? For what purpose did you use this plant? How did you use it? What part did you use? The popular names and descriptions of use were recorded.

A ranking method was used to identify the most culturally significant species, as this method has been shown to be highly useful in studies of local preferences [26]. The informants were asked to rank the species according to their preferences (using the figures).

### 2.3. Data Analysis

The nonparametric Kruskal-Wallis test was used to analyze the influences of gender, age, and education and the number of palms recognized and used by the informants. The analysis of variance was performed using BioEstat 5.0 software [37] and Excel was used for the correlation analysis (Windows 7).

The analysis was performed for the two age groups 18–40 and 41–82 [38–40]. The respondents' levels of education were categorized as illiterate, some elementary education, completed elementary education, some secondary education, completed secondary education and some higher education.

To analyze the rankings of the photos, salience was calculated using the ANTHROPAC software [41]. To analyze the knowledge and use of the palms, the obtained information was analyzed by ethnobotanical indexes according to the consensus among the informants [2, 42, 43] (Table 1). The values of the total species diversity (SDtot) and total species equitability (SEtot) were calculated to characterize the diversity in the use of palms and the uniformity of the degree to which the various species contribute to the daily lives of the informants.

## 3. Results and Discussion

The survey generated 1,928 records of use for the 16 palm species. The uses were grouped into 10 categories (Table 2) as follows food/direct use, food/cooking, food/animal feed, handicrafts, construction, medicinal, biofuel, toxic, ritual, and fertilizer.

### 3.1. Diversity of the Known and Used Palms

In the Kalunga community studied here, respondents recognized 16 palm species belonging to nine genera (Table 3). Approximately 51 common names were cited to designate the 16 species recorded (Table 3). All palms in this study are native to the Cerrado, and only *Syagrus oleracea* (guariroba) was cited as cultivated by one informant. *Euterpe edulis* (palmiteiro) was the only species identified that has a vulnerable conservation status [44].

The number of palms recognized (NPR) varied between 1 and 16 (mean 13) and the number of palms that have some use (NPU) varied between 5 and 16 (mean 13). The total species diversity (SDtot) for all the palms used by the Kalungas was SDtot = 15.47, while the value of total species equitability was SEtot = 0.97. These values indicate that many species were mentioned by many informants and that the palms are homogeneously used throughout the community. In another study about palms, Byg and Balslev [2] suggest a heterogeneous distribution of knowledge about palms, demonstrating that many palm species were used by only a few informants.

### 3.2. The Influence of Gender, Age, and Education on the Recognition and Use of Palms

Among women and men, the average numbers of palms recognized (NPR) were 12.5 (±3.3) and 12.9 (±3.8), respectively, and the average numbers of plants used (NPU) were 12.6 (±3.0) and 13.3 (±2.1), respectively. The NPR (*P* = 0.69) and NPU (*P* = 0.57) were not significantly different between men and women (Kruskall-Wallis test). However, the informant diversity index (IDs) shows a difference in knowledge between the genders. The average IDs for women was 34.46 (±9.2), while for men it was 21.69 (±4.8) with *P* < 0.05. Moreover, the average informant equitability index (IEs) was 0.73 (±0.19) for women and 0.8 (±0.18) for men.

Rural areas often feature stark divisions of labor, and the relationship between gender and plant knowledge seems to be correlated with the genders' different obligations to the community [45]. These authors point to the accumulation of roles for women, who, in addition to the “easier” work (housework, food preparation, gardening, etc.), also participate in the “heavy” work (planting and harvesting in the plantation).

The greater diversity of palm use among women can be explained by the large array of responsibilities that Kalunga women and other rural women take on to ensure the maintenance and survival of the family, especially the children. Activities such as obtaining firewood or long walks to arrive in the plantation areas are possible explanations for the higher exposure of women to different palm species. The highest value of equitability of use among men was associated with the category of construction, which is primarily performed by men.

Analyzing two age groups (18–40 and 41–82) revealed that the NPR values were 12.8 (±2.8) and 12.4 (±4.4), respectively, while the NPU values were 12.4 (±2.8) and 13.4 (±2.5), respectively. The analysis of variance (Kruskal-Wallis) showed no significant differences in the recognition (*P* = 0.52) and use (*P* = 0.07) of the species between the age groups (*P* > 0.05). However, in the second age group, the NPU is greater than the NPR. This result is explained by the nonrecognition of certain species by some older informants with vision problems. The use of visual stimuli among older informants had limitations. Previous studies using visual stimuli also observed this limitation [46].

Analyses correlating age and knowledge of plants, especially knowledge of medicinal uses, have shown that older age corresponds with greater knowledge about plants [47, 48]. Regarding the use of palms in the Engenho II community, age differences are not very pronounced. The noticeable familial relationship among community members strongly suggests that the family is mainly responsible for the temporal continuity of knowledge. The older people in the community are highly respected, and their homes are visited by younger relatives for several reasons. The young people, in turn, receive training from their elders and learn new ways of using the palms. Thus, the transmission and exchange of knowledge is not interrupted in this community. However, some products derived from palms are being replaced by industrialized products, such as oils, and as some practices are abandoned, knowledge of palms in the Engenho II will likely change over the long term.

In the case of the Engenho II community, education did not have a statistically significant effect on the NPR (*P* = 0.2) or the NPU (*P* = 0.75), the numbers of palms recognized and used by the informants (*P* > 0.05). Some authors assume that formal education decreases traditional knowledge [47, 49]; however, other studies do not observe this effect [50].

### 3.3. Distribution of the Knowledge and Use of Palms

**Figure 3:**
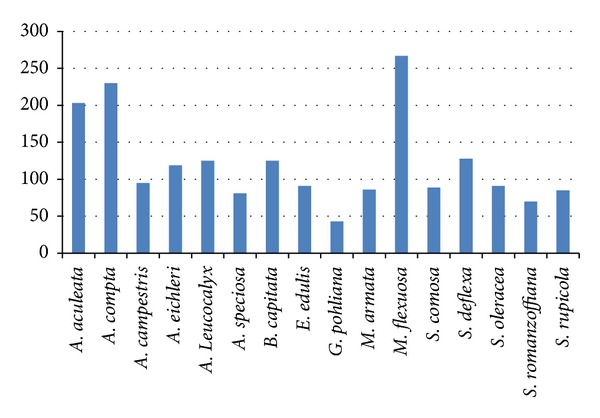
Number of citations of the use of palm species in the Engenho II community, Cavalcante, GO, central western Brazil. (*Acrocomia aculeate*,* Attalea compta, Allagoptera campestris, Attalea eichleri, Allagoptera leucocalyx, Attalea speciosa, Butia capitata, Euterpe edulis, Geonoma pohliana, Mauritiella armata, Mauritia flexuosa, Syagrus comosa, Syagrus deflexa, Syagrus oleracea, Syagrus romanzoffiana, and Syagrus rupicola*)*. *

Regarding the number of citations of use (*n* = 1928) by species, *Mauritia flexuosa* (buriti palm) was the best-represented species, followed by *Attalea compta *(indaiá) and *Acrocomia aculeata* (macaúba) (Figure 3 and Table 4).

#### 3.3.1. Food Use

Among the Kalungas of the Engenho II community, food use happens either directly (*in nature*) or in cooking, and all palm types of the area are used in this category. The mesocarp, epicarp, seeds, palm heart, and stipe of various species are used in both ways. In addition to *in natura* use, the mesocarp is used to prepare juices, desserts, milk, candies, and liquor (immersed with the fruits of *Butia purpurascens*).

*Acrocomia aculeata* had the greatest number of parts used for food (6). Many Kalungas extract oil from the seeds of some species for use in cooking and to make milk that is used in other preparations. The palm hearts of 13 species (*n* = 16) are consumed among the Kalungas of the Engenho II community.

Palms provide a variety of food sources for the Kalunga people. Frequently, palms are cited as an “emergency food,” referring to the consumption of fruits and seeds during the planting seasons and during the long walks between the plantation and the housing.

Some food uses are mentioned by the oldest individuals as “uses of another era,” from times when there was no food and palms provided an alternative way to end hunger. Using the toasted epicarp to make coffee and extracting starch from the pith of the stipe of *A. aculeata* are apparently no longer necessary.

The importance of food uses of palms has been noted in studies conducted in different regions [51–54]. Among the Kalungas of the Engenho II community, their survival has been linked to the use of palms as food. However, it is not possible to know whether the survival of palms is threatened by the community's continued use of parts that compromise the palms' survival (palm heart) and reproduction (seeds).

#### 3.3.2. Use in Handicrafts

Among the Kalungas of Engenho II, all palm species of the region (*n* = 16) were cited as used in handicrafts (Table 4). Different parts of the palms are cited in this category and appear to be useful in a direct way (“*in natura*”) or as raw materials for the manufacture of handicrafts. The use of the peduncular bract of species with underground stems as spoons and the sheaths of buriti palms as dustpans are examples of direct use.

The Buriti palm (*Mauritia flexuosa* L.f.) contributed the largest number of parts in this category (7; *n* = 18), and almost all utensils (quibanos, sieves, tapitis) present in residences are manufactured from this species. Details on the use of Buriti palm by the Kalungas were described by Martins et al. [10].

Handicraft production also identifies communities that represent themselves as ethnic or as Quilombo, as this is a source of income and cultural expression [55]. Among the groups of Quilombolas from the Amazon, materials derived from nature are commonly used for handicrafts, and palm species are one common source of these materials [55]. In the same region, the use of palms in handicrafts among indigenous and river communities is described in the works of Balick [56–59].

In the Cerrado of the Krahò indigenous territory (TO), approximately six of the 17 species of palms identified in the region provide raw material for handicrafts, which are sold in villages and neighboring towns [5, 53]. In another community of Tocantins, M. Sousa and A. Sousa [60] highlight the replacement of products manufactured with palms by more modern products, emphasizing the evidence that this substitution is related to the accelerated destruction of native vegetation, particularly in the valleys.

In the present study, while all species were used for handicrafts, most products are made with the Buriti palm petiole, which is collected under the plant after the leaves have fallen. This use is low impact and does not pose any risk to the plant. However, the uses of silk extracted from young leaves of the buriti palm and the stipe of the *Euterpe edulis* (palm heart) to manufacture beds and doors may have negative impacts on the populations of these species.

#### 3.3.3. Use in Construction

Among the Kalungas, 11 species are used for construction, with *Mauritia flexuosa* (buriti palm) and *Attalea compta *(indaiá) being the most frequently cited in this category, followed by *Attalea eichleri* (Pindoba), *Euterpe edulis *(palm heart), *Attalea speciosa* (coco-palmeira), and *Mauritiella armata* (buritirana). The leaves of the buriti palm and indaiá are used together to cover houses. These leaves are gathered under the waning moon but never during the new moon, because according to local tradition, this is the best time to collect them to avoid infestations of insects and fungi in the leaves and to produce more durable structures. In contrast, Shanley and Rosa [61] observed that among the Caboclos of Pará, it is said that the best time to collect the leaves is actually under the new moon because the leaves will be less damaged by insects at this time.

The use of palms for construction has been observed in ethnobotanical studies in various regions of the country [5, 52, 54, 56, 62], with leaves and stipe as the preferred parts for this category.

#### 3.3.4. Medicinal Use

The Kalungas of Engenho II cited medicinal use for eight species (Table 5). The following parts are used for medicinal purposes: stipe, leaf (petiole, rachis, and whole leaf), fruits (mesocarp, endocarp, liquid endosperm, and seed), roots, and palm hearts.

Mesocarp oil was the most frequently cited medicinal part, followed by the seed oil, palm heart, and root. The main therapeutic indication of the palms was for the treatment of respiratory diseases such as flu and pneumonia. The second most common use is against snake bites. Two species share these two uses, the Buriti palm (*Mauritia flexuosa*) and Macaúba (*Acrocomia aculeata*). Two species were used for tooth pain, Macaúba and Indaiá (*Attalea compta*).

The importance of palms in medicine and pharmacology is discussed by Sosnowska and Balslev [63]. Based on a review of the literature over the last 25 years, these authors identified 106 species of palms in the Americas with medicinal uses. In this study, the fruit was the most frequently used part for medicinal purposes (56 spp.), followed by oil (19 spp.), mesocarp (16 spp.), and the endosperm (11 spp.). Other parts of the palm were also mentioned, including the root (27 spp.), the leaves (22 spp.), the palm heart (19 spp.), the stipe (17 spp.), and flowers (9 spp.).

#### 3.3.5. Other Uses: Ritual, Fertilizer, Biofuel, and Toxic Uses

Among the Kalungas, of the three species placed in the ritual category, two (*Euterpe edulis* and *Geonoma pohliana*) were used to create decorative “*lapinhas,*” large bows used for the closure of a traditional religious festival. In this festival, the plants are placed at the front of the church such that the people pass underneath the plants before entering into the religious space. *Butia purpurascens* leaves were cited by one informant as used for incense.

In the Amazon region, one species of palm (*Socratea exorrhiza*) has previously been described as having a ritual use [54]. Among the Krahò Indians, two species are in this category (*Mauritia flexuosa*, buriti palm, and *Oenocarpus distichus, *bacaba) [5]. The Buriti stipe is used in traditional log races, a practice observed in different indigenous ethnic groups in the central western region of Brazil.

Decomposed Buriti palm pith and stipe were cited as used for fertilizer (manure) by three informants. Use as a biofuel has been cited for four species. The leaves of three species (*Butia purpurascens*, and *Syagrus deflexa, Syagrus oleracea*) are used to light fires, and the dried fruit of *Attalea compta *(indaiá) is used as charcoal.

Three palm species were cited as toxic by five informants. Consumption of mesocarp and seedsof *Allagoptera leucocalyx* (licuri-rasteiro-da-mata), *Butia purpurascens *(cabeçudo), and *Syagrus deflexa* (licuri-da-serra) are contraindicated because they cause stomach pain. The liquid of the mesocarp pulp of the licuri-da-serra is also contraindicated for people with respiratory diseases.

### 3.4. The Importance of Palms

The most prominent plant for the Kalunga people of the Engenho II community is *Mauritia flexuosa* (buriti palm), followed by *Attalea compta *(indaiá), *Acrocomia aculeata* (macaúba), *Butia purpurascens* (cabeçudo), *Attalea eichleri* (pindoba), and *Syagrus comosa *(gariroba-catolé). The buriti palm was the first in 88% of the rankings and was present in all of them (100%) (Table 6).

The average importance value in this study was 0.35 (±0.3), with five species showing values above 0.49. Comparing the results of importance values (IVs) with the analysis of salience, the most important species are the same in both analyses (Table 6), and consequently, no significant differences in the results were observed between the methods (*P* = 0.1).

Byg and Balslev [2] found differences in the importance and use of palms between species, indicating that specific characteristics determine how a species is used and appreciated. According to these authors, many ethnobotanical studies assume that the importance of a plant is related to the ways that it is used. To test this premise, we calculated the correlation coefficient between the salience and the number of different uses of the palm species mentioned in the study. The results indicated a moderate to positive correlation (*r* = 0.64), demonstrating that the more uses a palm has, the greater its cultural importance to the Kalungas of the Engenho II community is.

### 3.5. Richness and Distribution of Knowledge

Ethnobotanical indexes for the richness and distribution of knowledge among palm species were calculated (Table 7). The average use diversity (UDs) was 2.47 (±1.29), and the use equitability (UEs) was 0.52 (±0.26). The results indicate that the uses of species are distributed almost uniformly among categories.

Comparing the results of IVs and salience with those of the use diversity (UDs), three of the six most important species showed the highest values of this index. However, the species with the lowest IVs (*Geonoma pohliana*) had the fourth highest use diversity index. Considering the number of different uses for each species, the buriti palm (*Mauritia flexuosa*) and cabeçudo (*Butia purpurascens*) had the greatest diversity in categories of use (7; *n* = 10). However, *Butia purpurascens *showed a low UD value (2.15), as it was almost exclusively used for making brooms.

Regarding the distribution of uses among species, the values of informant diversity indexes (IDs) indicate that the species with the greatest diversities of use were *Attalea compta *(indaiá) (74.51), *Allagoptera leucocalyx* (licuri-rasteiro-da-mata) (74.05), and *Mauritia flexuosa* (buriti) (67.7). Byg and Balslev [2] identified a positive correlation between the informant diversity (Ids) and the importance of the palm, which was also observed in the current study. The relatively high average value of the informant equitability (IEs: 0.75) indicates that almost all people who know a particular species know relatively the same number of uses (Table 7). The average use consensus among informants (UCs) was 0.68 (±0.3). This indicates that expressive agreement exists among informants for most species.

On the other hand, the average purpose consensus (PCs) value, which measures the agreement of the informants regarding a specific purpose, was very low (Table 7). This low value indicates that the informants use the same species for different categories of use. Although it had a high importance value and many use citations, the buriti palm (*Mauritia flexuosa*) had the lowest PC value, demonstrating a low degree of overlap between informants and that many uses of buriti palm are not very popular, such as cosmetic use, which was mentioned only once. Additionally, statements by the oldest individuals, such as “this was done in difficult times to obtain food to eat,” show that some uses have been abandoned.

On average, the informants know 12.65 (±3.5) species and use 12.84 (±2.7) (*n* = 16). The numbers of informants who recognize and use all species of palms are 19 (*n* = 88) and 14 (*n* = 88), respectively. Based on the 10 use categories in the present study, the average number of categories of use of a species was 4.9 (±1.2). The average values of species diversity (SDi) and equitability (SEi) were 10.02 (±2.3) and 0.73 (±0.2), respectively. These values suggest that knowledge about the use of palms is relatively high and is well distributed throughout the community.

### 3.6. Use and Value of Various Palm Parts

The analysis of the palm parts considered all 18 parts that were cited. However, only the results from the five most important parts are presented here (Table 8). The plant part value (PPV) is a value given to a specific part of the plant.

The fruit was the most frequently cited part in each palm species. The seed and mesocarp were frequently cited for the food category. The fruits of 16 palm species have been described as useful by the local population. All species with a pulpy mesocarp are consumed *in natura* or used in the manufacture of desserts and in other types of cooking. The following species are highlighted in this group: *Acrocomia aculeata* (macaúba), *Mauritia flexuosa* (buriti), *Syagrus romanzoffiana* (jarobá) and *Allagoptera leucocalyx* (Licurí-rasteiro-da-mata).

The palm seeds are heavily used by the Kalungas of the Engenho II community. Many species provide seeds that are eaten *in natura*, such as *Acrocomia aculeata* (macaúba), *Allagoptera campestris* (licurí-rasteiro-do-campo), *Allagoptera leucocalyx* (licurí-rasteiro-da-mata), *Syagrus deflexa* (licurí-da-serra) and *Syagrus rupicola* (catolé-da-serra). Another group of species have seeds that are also used to extract oil: *Attalea speciosa* (coco-palmeira), *Attalea compta *(indaiá), and *Attalea eichleri* (pindoba).

Informants also described uses for whole leaves, with *Butia purpurascens* (cabeçudo) exhibiting the highest value for this part, followed by *Mauritiella armata* (buritirana), *Attalea compta *(indaiá), and *Mauritia flexuosa* (buriti palm) (Table 8). The leaves are used for construction and for the manufacture of handicrafts and household items. The species with the highest extraction is *Butia purpurascens*. Among the Kalungas and in other regions where it occurs, its leaves are used to make domestic brooms [64]. However, in the Kalunga region, groups of pickers visit the region annually to collect large amounts of leaves. This intense and disordered extraction may be causing serious damage to the populations of this species, which tend to be aggregated in easily accessible locations.

The stipe was used in nine species, including two forest species known to the Kalungas, *Euterpe edulis* and *Geonoma pohliana *subsp. *weddelliana *(Table 8). The stipes of these two species were mentioned by the oldest respondents as a “former” use for building roofs and beds. The fact that *Euterpe edulis* is included in the endangered species list in Brazil [65] clearly shows the need for an updated and well-reasoned approach to this subject within the community.

The palm heart was a significant part, with 14 species providing useful palm hearts (Table 8). However, the *Syagrus oleracea* (gariroba-verdadeira) and *Syagrus comosa* (gariroba-catolé) palm hearts are preferred for consumption, as both have a bitter taste that is highly appreciated by the Kalungas. Unfortunately, these two species have solitary stipes, and extracting this part kills the palm. Throughout this research, few individuals of *S. comosa* were observed near the community, and the only two observed individuals of *S. oleracea* were growing in the backyard of a residence. Some informants were concerned about possible punishment from environmental agencies because of this practice, as demonstrated in the following sentence: “we pick them up, but not too much because if the *zimbamba* see it, we will have problem.” Zimbamba refers to individuals from the Chico Mendes Institute, formerly called the IBAMA (Brazilian Institute of Environment and Renewable Natural Resources).

### 3.7. Final Considerations

- All of the palms present in the region of community Kalunga Engenho II were cited as useful by the informants. Species occur in different vegetation types: rupestrian Cerrado, gallery forest, grassland, shrub savanna, and valleys.
- Palms were most frequently used for food, handicrafts, and construction, demonstrating that the use of palms by the Kalunga people did not differ from the categories recognized in other studies performed in the Cerrado and in the Amazon. All species of palms of the area were cited as useful for food and handicrafts, demonstrating their importance for the survival of the Kalungas over time. The medicinal category featured the largest number of different parts.
- The variables gender, age, and education did not affect the numbers of plants recognized and used by the Kalungas. However, the informant diversity and equitability indexes show that women know more different uses than men, although men's knowledge is distributed more homogeneously.
- The indices calculated to evaluate the distribution of the knowledge among the Kalungas demonstrated that knowledge is well distributed throughout the community. The most important palm for the Kalungas of the Engenho II is *Mauritia flexuosa* (buriti palm). This species occurs in the vicinity of the community and was recognized by all informants. Despite the high number of use citations for this species, the diversity use index was low, demonstrating that the uses are virtually the same among informants.
- The palm fruits represent an important source of vitamins and protein for the local population, and all species (*n* = 16) are sources of food. The use of oil seeds was widely cited among the informants, but in many homes, it is now being replaced by commercial oils. Although many residents have knowledge about the practice of extracting oils, all oils identified in the residences come from other, more distant Kalunga communities.
- Use of leaves predominantly occurred in two species, *Mauritia flexuosa* (buriti palm) and *Attalea compta *(indaiá). The leaves are extracted due to spontaneous demand, in accordance with indications of traditional knowledge. Based on a study conducted in the Jalapão region (TO-state), Sampaio et al. [9] suggest guidelines for the sustainable extraction of buriti palm leaves. That literature will be incorporated into a “Guide of the palms from the Engenho II,” which is in preparation.
- The palm hearts of 14 species (*n* = 16) were consumed, primarily for family consumption and for festivities in the community. Until now, it was not possible to know whether the extraction of palm hearts was related to any business. This delicacy is special due to its taste and its importance to the welfare of the Kalunga people. The use of the apical meristem, apparently in response to spontaneous demand, is believed to be affecting the abundance of some species of palms near this community. This finding demonstrates the need for studies related to the phenology, phytosociology, and management of species with bitter palm hearts, the variety preferred by this community.

## Figures and Tables

**Table 1:** Indexes used to analyze the knowledge and use of palms in the Kalunga Community Engenho II, Cavalcante, GO, central western Brazil [2, 42, 43].

Indexes	Calculation	Description
Total species diversity (SDtot)	SDtot = 1/*∑Ps* ^2^ (*P* = total contribution of species *s* to the total use; number of times species *s* was cited divided by the total number of citations).	Measures how the species are used and how they contribute to the total palm use. Values range from 0 to *n*.
Total species equitability (SEtot)	SEtot = SDtot/*n* (*n* = number of species used).	Measures how different species contribute to total palm use independent of the number of species used. Values range from 0 to 1.
Importance value (IVs)	IVs = nis/*n* (number of informants who considered species *s* the most important). *n* = total number of informants.	Measures the proportion of informants who cited one species as the most preferred. Values range from 0 to 1.
Use diversity values (UDs)	UDs = 1/SPc² (Pc = contribution of the use category *c* to the total utility of species *s*; the number of times species *s* was mentioned within each use category divided by the total number of citations of use of species *s* among all use categories).	Measures how a species is used within a category and the degree to which it contributes to the overall use of this species. The values range from 0 to the number of categories of use for which the plant is used.
Use equitability value (UEs)	UEs = UDs/UDsmax (UDsmax = the maximum use diversity value for a species with a given number of categories).	Measures how different uses contribute to the overall use of a species, independent of the number of categories of use. Values range from 0 to 1.
Informant diversity value (IDs)	IDs = 1/*∑P*_*i*_^2^; *P*_*i*_ = contribution of informant *i* to the total knowledge of species *s* (number of uses mentioned by an informant divided by the total number of uses).	Measures how many informants use one species and how its use is distributed among informants. Values range from 0 to the number of informants that use the species.
Informant equitability value (IEs)	IEs = IDs/IDsmax (IDsmax = the maximum diversity value of an informant for species *s*).	Measures how the use of a species is distributed among informants, regardless of the number of informants who use it. The values range from 0 to 1.
Use consensus value (UCs)	UCs = 2ns/*n* − 1 (ns = number of people who use species *s*).	Measures the degree of agreement among informants regarding whether a species is useful. Values range from −1 to +1.
Purpose consensus value (PCs)	PCs = *∑* Pu²/S (Pu = proportional contribution of the use *u* to the overall utility of the species *s*; S = total number of different types of use of species *s*). PCs represents the number of times that use *u* has been recorded for species *s* divided by the total number of recorded uses for species *s*.	Measures the degree of agreement between informants regarding a given use. Values range from 0 to 1.
Species diversity value (SDi)	SDi = 1/*∑* Ps² (Ps = contribution of a species *s* to the total use of a species by informant *i*; the ratio between the number of times that the informant mentions a species divided by the total number of responses of the informant).	Measures how an informant uses many species and how the uses are distributed among species. Values range from 0 to the number of species used by the informant.
Species equitability values (SEi)	SEi = SDi/Sd imax (Sd imax = maximum value of species diversity for informant *i*)	Measures how an informant uses known plants, regardless of the number of plants used. Values range from 0 to 1.
Part of the plant value (PPV)	The ratio between the total number of reported uses for each plant part (*∑*RU_plant_ _part_) and the sum of uses reported for that plant (*∑*RU).	Indicates the difference in the number of uses of the parts of the plant and reveals the most frequently used part of the plant.

**Table 2:** Descriptions of the 10 categories of use identified for palms in the Kalunga community Engenho II, Cavalcante, GO, central western Brazil.

Category of use	Description
Food/direct use	The consumption of fruits or other plant parts occurs *in natura*.
Food/cooking	The part used (fruit, seed, palm heart) is manipulated for the consumption of juice, cake, dessert, porridge, oil, and meal.
Food/animal feed	Used to feed domestic animals.
Handicrafts	Household items (“tapiti,” sieve, “quibano”), rustic furniture (sofa, bookshelf, table), decorative objects, and direct use of any part of the plant (e.g., using the sheath of a buriti palm as a dustpan).
Construction	Construction and/or coverage of ranches, houses, kitchens, hen houses, and pigsties.
Medicinal/cosmetic	Use of any part of the palm, alone or manipulated with other species for the manufacture of homemade medicine and cosmetics (only one cosmetic use was cited).
Ritual	Decoration in religious ceremonies and blessings.
Fertilizer	Use of decomposed plant parts as fertilizer.
Biofuel	Use of dried leaves to light fires and of dried fruits as charcoal.
Toxic	Citations of contraindicated uses.

**Table 3:** List of species, common names, parts used, and the uses of palms in the Kalunga community Engenho II, Cavalcante, GO, central western Brazil. In the “Vernacular names” column, the bolded names indicate those most frequently used by the informants; in the “Uses” column, the number indicates the number of different uses for the species.

Scientific name	Vernacular names	Parts used	Uses
*Acrocomia aculeata* (Jacq.) Lodd. ex Mart.	Macaúba Palm, xodó	Fruit (epicarp, mesocarp, seed), stipe (“shell" and center core), palm heart	(5) Food (direct use, cooking and animal feed), handicrafts, medicinal
*Allagoptera campestris* (Mart.) Kuntze	Licuri rasteiro do campo, licurizinho, coquinho rasteiro, licurizinho do cerrado, coquinho de licuri	Leaf, fruit (mesocarp, seed), palm heart	(3) Food (direct use, cooking), handicrafts
*Allagoptera leucocalyx* (Drude) Kuntze	Licuri rasteiro da mata, coco licuri, licurizinho, licuri rasteiro, licurizinho rasteiro, coquinho painha	Mesocarp, seed, palm heart	(6) Food (direct use, cooking), handicrafts, construction, medicinal, toxic
*Attalea compta* Glassman	Indaiá, daiá, babaçu	Fruit (mesocarp, endocarp, seed), leaf, palm heart, peduncular bract, infructescence	(6) Food (direct use, cooking), handicrafts, construction, medicinal, fuel
*Attalea eichleri* (Drude) A.J. Hend.	Pindoba, palhinha, piaçaba, painha, coco-painha, candoba	Fruit (mesocarp, seed), leaf, peduncular bract	(5) Food (direct use, cooking), handicrafts, construction, medicinal
*Attalea speciosa* Mart. ex Spreng.	Palm, coco-palmeira, babaçu	Leaf, fruit (endocarp, mesocarp, seed), palm heart	(4) Food (direct use, cooking), handicrafts, construction
*Butia purpurascens* (Mart.) Becc.	Cabeçudo	Leaf, fruit (mesocarp, seed), peduncular bract, inflorescence, palm heart	(7) Food (direct use, cooking), handicrafts, medicinal, ritual, biofuel, toxic
*Euterpe edulis* Mart.	Palmito, palmito-do-brejo, açaí	The whole plant, stipe, palm heart, fruit (mesocarp, seed), leaf, peduncular bract	(5) Food (direct use, cooking), handicrafts, construction, ritual
*Geonoma pohliana* subsp. *weddelliana *	Palmita, palmito-merim	The whole plant, stipe, mesocarp, seed	(5) Food (direct use, cooking), handicrafts, construction, ritual
*Mauritia flexuosa* L.f.	Buriti palm	Fruit (epicarp, mesocarp, seed), leaf (the whole leaf, sheath, petiole, new unopened leaf), stipe, root	(7) Food (direct use, cooking, and animal feed), construction, handicrafts, medicinal, cosmetic
*Mauritiella armata* (Mart.) Burret	Buritirana, buriti-merim, pati	Stipe, palm heart, leaf (the whole leaf, petiole), root, fruit (mesocarp)	(5) Food (direct use, cooking), handicrafts, medicinal, construction
*Syagrus comosa* (Mart.) Mart.	Garioba catolé, garioba, garioba-do-campo, garioba da serra, gariroba-catolé, coco-catolé, gariobina-do-cerrado, gueiroba, gariobinha, catolezinha, garioba-comum	Palm heart, fruit (mesocarp, endocarp, seed), stipe	(5) Food (direct use, cooking), handicrafts, construction, medicinal
*Syagrus deflexa * Noblick and Lorenzi	Licuri da serra, coquinho-do-cerrado, licurizinho-da-serra, coquinho-de-licuri, coquinho-da-serra, paia-de-nicuri	Leaf, fruit (mesocarp, endocarp, seed), palm heart	(5) Food (direct use), handicrafts, construction, biofuel, toxic
*Syagrus oleracea* (Mart.) Becc.	Garioba verdadeira, gariroba verdadeira, gueroba	Peduncular bract, fruit (mesocarp, endocarp, seed), palm heart	(4) Food (direct use, cooking), handicrafts, biofuel
*Syagrus romanzoffiana* (Cham.) Glassman	Jarobá	Fruit (mesocarp, seed), palm heart, inflorescence	(4) Food (direct use, cooking, and animal feed), handicrafts
*Syagrus rupicola* Noblick and Lorenzi	Catolé, catolé-rasteiro, catolé-da-serra, catolezinho, licuri-de-raposa, catolezinho, coquinho-catolé, coquinho-da-serra	peduncular bract, mesocarp, seed, palm heart	(3) Food (direct use, cooking), handicrafts

**Table 4:** Number of citations, species, and parts used for each category of palm use among the Kalunga from the Engenho II community, Cavalcante, GO, central western Brazil.

Category of use	Number of citations (*n* = 1928)	Number of species used (*n* = 16)	Number of parts used (*n* = 18)
Food/cooking	444	15	6
Food/animal feed	12	3	4
Food/direct use	889	16	8
Handicrafts	264	16	12
Biofuel	8	4	2
Construction	240	11	4
Fertilizer	3	1	1
Medicinal/cosmetic	60	8	10
Ritual	19	3	3
Toxic	6	3	2

**Table 5:** Species cited as medicinal, number of citations, parts used, and therapeutic indications, Engenho II community, Cavalcante, GO, central western Brazil.

Species (number of citations)	Part used	Therapeutic indication
*Acrocomia aculeata* (17)	Fruit (endocarp oil)	Toothache, ear diseases
	Fruit (seed oil)	Ear diseases
	Palm heart (juice)	Antivenom
*Allagoptera leucocalyx* (2)	Palm heart (juice)	Ear and digestive system diseases
*Attalea compta* (8)	Fruit (endocarp oil, mesocarp, and seed)	Toothache
*Attalea eichleri* (3)	Leaf (rachis juice)	Skin diseases (burning)
	Fruit (liquid endosperm)	Ocular diseases
*Butia purpurascens* (1)	Fruit (mesocarp)	Skin diseases (healing)
*Mauritia flexuosa* (26)	Leaf (petiole juice)	Antivenom
	Fruit (mesocarp oil)	Antivenom, cardiovascular, and respiratory diseases
	Fruit (toasted seed)	Reproductive system diseases
	Root	Musculoskeletal and rheumatic diseases
*Mauritiella armata* (6)	Stipe	Skin diseases (burning)
	Root	Genitourinary system and rheumatic diseases
*Syagrus comosa* (2)	Palm heart (juice)	Digestive system diseases

**Table 6:** Salience analysis calculated from the rankings of palm species by the Quilombo Kalunga of the Engenho II community, Cavalcante, GO, central western Brazil (in order of salience). IVs: importance values.

Species	Frequency (%)	Mean	Salience index	IVs
*Mauritia flexuosa *	100.0	1.70	0.882	0.977
*Attalea compta *	86.0	2.78	0.599	0.841
*Acrocomia aculeate *	81.4	3.09	0.527	0.795
*Butia purpurascens *	67.4	4.66	0.257	0.659
*Attalea eichleri *	50.0	4.05	0.242	0.489
*Syagrus comosa *	33.7	3.66	0.181	0.329
*Euterpe edulis *	32.6	4.71	0.124	0.239
*Attalea speciosa *	29.1	3.88	0.151	0.284
*Syagrus oleracea *	26.7	4.13	0.126	0.261
*Allagoptera leucocalyx *	24.4	4.48	0.103	0.239
*Syagrus deflexa *	18.6	4.69	0.072	0.182
*Mauritiella armata *	16.3	4.00	0.077	0.170
*Syagrus rupicola *	8.1	4.00	0.039	0.079
*Syagrus romanzoffiana *	8.1	4.14	0.039	0.079
*Allagoptera campestris *	1.2	6.00	0.002	0.011
*Geonoma pohliana* subsp. w*eddelliana *	1.2	6.00	0.002	0.011

**Table 7:** Summary of the quantitative values capturing the uses and importance of palm species among the Kalunga of the Engenho II community, Cavalcante, GO, central western Brazil.

	Mean value (min., max.)	Standard deviation
Per palm species		
Number of citations	1928	
Number of categories	10	
Use diversity (UDs)	2.47 (1.04, 4.8)	1.29
Informant diversity (IDs)	58.85 (32.67, 75.26)	13.76
Informant equitability (IEs)	0.77 (0.42, 1)	0.18
Use consensus (UCs)	0.68 (−0.09, 1)	0.30
Purpose consensus (PCs)	0.12 (0.03, 0.39)	0.08
Per informant		
Number of informants	88	
Number of categories	5 (3, 7)	1.15
Number of species used	12.84 (5, 16)	2.72
Species diversity (SDi)	10.02 (4.26, 13.71)	2.33
Species equitability (SEi)	0.73 (0.31, 1)	0.17

**Table 8:** Value of use index of the different parts (PPV) of palm species assigned by the Kalunga community Engenho II, Cavalcante, GO, central western Brazil.

Species	Stipe	Leaf	Mesocarp	Seed	Palm heart
*Acrocomia aculeate *	0.02	—	0.43	0.45	0.05
*Allagoptera campestris *	—	0.01	0.25	0.72	0.01
*Allagoptera leucocalyx *	—	0.02	0.31	0.65	0.02
*Attalea speciosa *	0.01	0.24	0.05	0.67	—
*Attalea compta *	0.00	0.36	0.04	0.49	0.06
*Attalea speciosa *	—	0.22	0.05	0.65	0.04
*Butia purpurascens *	—	0.60	0.16	0.14	0.02
*Euterpe edulis *	0.51	0.01	0.24	0.07	0.09
*Geonoma pohliana *	0.30	—	0.30	0.07	0.09
*Mauritia flexuosa *	0.06	0.29	0.46	0.02	—
*Mauritiella armata *	0.21	0.49	0.17	0.01	0.01
*Syagrus comosa *	0.01	0.03	0.02	0.11	0.80
*Syagrus deflexa *	—	0.04	0.27	0.57	0.07
*Syagrus oleracea *	0.03	0.01	0.02	0.29	0.59
*Syagrus romanzoffiana *	—	—	0.56	0.30	0.07
*Syagrus rupicola *	—	—	0.18	0.75	0.04

Average	0.08	0.16	0.20	0.36	0.13
Standard deviation	0.15	0.20	0.17	0.27	0.24
